# A cross-sectional study on the burden and impact of migraine on work productivity and quality of life in selected workplaces in the Philippines

**DOI:** 10.1186/s10194-020-01191-6

**Published:** 2020-10-27

**Authors:** Nel Jason Haw, Ian Theodore Cabaluna, Germaine Erika Kaw, Joanna Feliz Cortez, Maria Pamela Chua, Kristel Guce

**Affiliations:** 1 Health Sciences Program, School of Science and Engineering, Ateneo de Manila University, Quezon City, Philippines; 2 Wellbridge Health Inc., Manila City, Philippines; 3 Novartis Healthcare Philippines, Inc., Makati City, Philippines

**Keywords:** Migraine, Burden of disease, Cost of illness, Quality of life, Health care utilization, Philippines, Workplace

## Abstract

**Background:**

Migraine imposes a substantial personal and economic burden to many working age individuals. This study aimed to evaluate the burden and impact of migraine on work productivity in selected workplaces in the Philippines.

**Methods:**

A cross-sectional survey was conducted among employees suspected or diagnosed with migraine February to May 2020. Volunteer employees were screened for migraine using the ID-Migraine™ test. Eligible employees were tested for migraine severity and impact on work productivity using the Migraine Disability Assessment (MIDAS) questionnaire. Quality of life was measured using the Short Form-36 (SF-36) questionnaire and additional questions on triggers, coping mechanisms, workplace assistance, and health care utilization were asked. Multiple logistic regression was used to identify significant predictors of migraine disability (high – MIDAS Grade III/IV vs. low – MIDAS Grade I/II). Differences in quality of life scores by migraine disability were measured using multiple linear regression. Productivity costs lost to migraine disability were calculated as the number of days lost to migraine multiplied by the self-reported wage rate, and costs according to migraine severity were measured using a two-part generalized linear model.

**Results:**

From around 24,000 employees who were invited to participate in the survey, 954 respondents provided consent and attempted to respond to the survey resulting to a response rate of around 4.1%. A total of 511 positive migraine screens were included in the final sample. Females comprised two-thirds of all positive migraine screens and were more likely to have high migraine disability (odds ratio: 1.60, 95% CI: 1.03–2.49) than males. Those with high migraine disability scored lower on role limitations compared to those with low migraine disability. Stress and looking at computer screens were cited as the top trigger for migraine, while sleeping enough hours and getting a massage were cited as top coping mechanisms. Three in four (77%) visited their company clinic within the past 3 months, which meant that most doctors seen for migraine-related symptoms were general practitioners. Five in six (85%) took medication for migraine, almost all of which were over-the-counter medications. Mean annual productivity costs lost due to migraine disability were PHP27 794 (USD556) per person.

**Conclusion:**

Migraine poses a significant threat to work productivity in the Philippines. Many opportunities, such as disease management and introduction of alternative options for migraine treatment, may be introduced to help address these issues.

## Introduction

Migraine is a neurological disorder recognized as one of the leading causes of disability in the world, estimated to impact anywhere between one in ten [1] and one in six individuals [2]. While several population-level studies on migraine have been conducted in the past few years, there remain countries with little information on the burden of migraine, specifically in the Asia-Pacific region [2]. A systematic review and meta-analysis on chronic migraine found only seven population-level studies in the Asia-Pacific region estimating chronic migraine prevalence to be approximately 6–17 people per 1000 population [3]. In the Philippines, the last known national-level migraine prevalence survey was in 2003, which found that 7.9% of the population screened positive for migraine [4]. However, in 2017, the Institute for Health Metrics and Evaluation (IHME) reports that headache disorders which include migraine has a prevalence estimate of about 17.3% [5].

Migraine is associated with significant impact on daily living, such as work, school, and personal activities [6]. Migraine patients consistently report poorer quality of life scores than healthy individuals on aspects of physical well-being, while chronic migraine patients consistently report poorer quality of life scores than episodic migraine patients on aspects of emotional well-being [7]. Additionally, the expectation of worry on the next migraine attack is in itself negatively affecting work productivity and quality of life [8].

Sex and age are significantly associated with migraine burden, with females at least twice as likely to report having migraine [1] and working-age individuals more likely to report having migraine than younger or older individuals [9]. This implies that migraine poses a significant economic burden and various studies in the past have tried to quantify the economic impacts of migraine. The European Eurolight project estimated that more than 90% of economic losses associated with migraine were attributable to indirect costs such as sick days and reduced work productivity as compared to less than 10% of direct costs such as medicines and outpatient consultation [10]. A systematic review from the United States found that on average around 2 to 3 workdays per month were lost due to migraine, with women reporting twice more workdays affected than men [11]. A Malaysian study found similar results with mean days affected by migraine being 5.6 days over the past 3 months among banking sector employees, with monetary losses potentially reaching as much as USD3000 annually for those with the most severe forms of migraine [12]. In a literature search, this was the only published study found to present data on the burden of migraine in the Asia Pacific region.

Given this, the present study assessed the burden and impact of migraine and work productivity and daily activities in selected workplaces in the Philippines. Focusing on those having migraine, this study explored the migraine patient journey regarding the frequency and severity of migraine attacks, triggers in the workplace, workplace assistance programs, availability and utilization of health care services, and quality of life. Finally, this study also measured monetary estimates on lost productivity.

## Methods

### Study design and setting

**Fig. 1:**
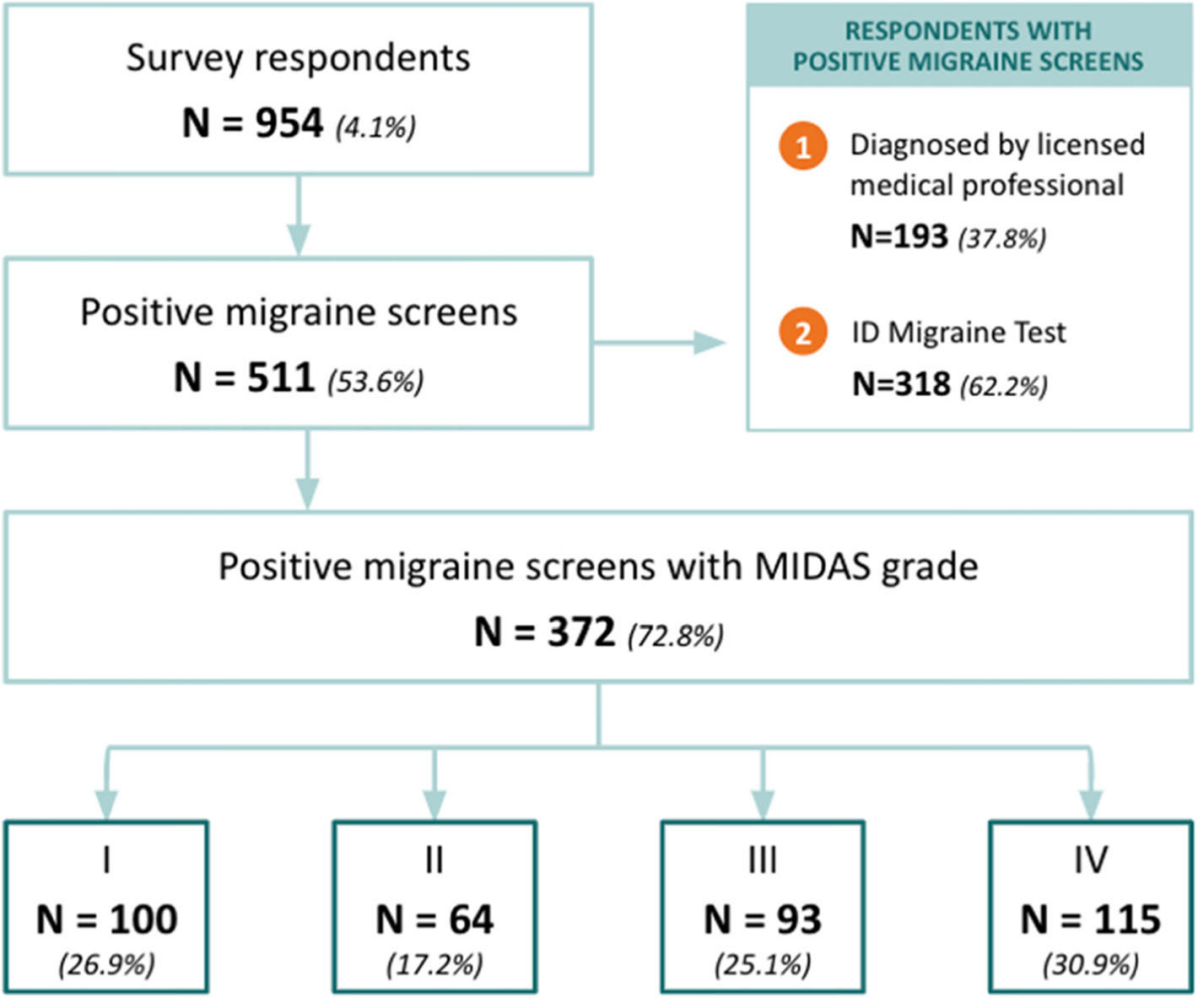
Study population flowchart

A cross-sectional study was conducted among employees of 12 companies in the Philippines across various industries, such as telecommunications, holdings groups, business process outsourcing, and finance, from February to May 2020. The survey was conducted online and through physical booths at company clinics, depending on the preference of human resources departments of the participating companies. All employees in these companies, estimated at 24,000, were invited to participate in the survey. To maximize survey participation rate, companies were selected based on the following criteria: having more than 50% share of females in the workforce, having generous employment benefits such as private health insurance and medicine allowances, and known for having a tough environment that may trigger migraine attacks, such as prolonged computer usage (Fig.1).

All interested participants in the survey underwent an informed consent process. In the online version of the survey, the first page introduced a short and long version of the informed consent form, and participants had three options to proceed: provide consent and proceed with the questionnaire, decline to participate and the survey ended, and a free text space to ask any clarificatory questions about the survey, with a member of the research team providing a response within 24 h via short messaging service (SMS). Since mobile phones are not registered to specific individuals and geographic locations in the Philippines, this ensured that participants remain anonymous while still reachable by the survey team to answer their questions.

Those who provided consent then answered a series of eligibility and migraine screening questions. Those eligible to answer the survey were permanent residents of the Philippines, who were age 18 or older at the time of the survey and worked at least 40 h a week in their company. Those who self-reported as having been diagnosed with migraine by a licensed medical professional, and those who screened positive using the ID Migraine™ Test, were considered as positive migraine screens and comprised the study sample who answered the full questionnaire. The ID Migraine™ Test consists of three questions on commonly reported migraine symptoms – nausea, limited ability to do daily tasks, and photosensitivity – and at least two symptoms must be reported for a positive screen. The test has been validated in multiple contexts and has been used in previous migraine population-level studies because of its simplicity and accuracy compared to a clinical diagnosis [13–15]. A systematic review found that the pooled sensitivity of the test is 84% (95% confidence interval (CI): 75% - 90%) and specificity is 76% (95% CI: 69% - 83%) [13].

### Questionnaire and study variables

The questionnaire was divided into the following sections [1]: socio-demographic information [2]; the Migraine Disability Assessment (MIDAS) Questionnaire, which measured migraine severity across four grades [3]; the Short-Form 36 (SF-36) Questionnaire, which measured quality of life across eight domains [4]; health care utilization, which looked at outpatient consultations, emergency room (ER) visits, hospitalizations, and medicines utilization; and [5] triggers and coping mechanisms in the workplace against a suspected migraine attack; and [6] assistance provided in the workplace, which asked actual support mechanisms and perceived usefulness of a wide range of support mechanisms, regardless of their availability in the workplace.

The burden of migraine was measured as the MIDAS Score, which is measured as the total number of workdays lost to migraine over a three-month period. The MIDAS Score may be coded into MIDAS Grades, indicating migraine severity: Grade I (0 to 5); Grade II (6 to 10); Grade III (11 to 20); and Grade IV (21 and above). The MIDAS Score may also be disaggregated into two: number of workdays lost due to migraine, also known as absenteeism; and number of workdays with impaired work productivity due to migraine, also known as presenteeism. Daily costs attributable to absenteeism were calculated as absenteeism multiplied by the daily rate, while daily costs attributable to presenteeism were calculated as presenteeism multiplied by the daily rate. The daily rate was calculated as the self-reported gross monthly income divided by 22 working days. Annual economic costs of migraine were calculated as the daily costs attributable to absenteeism and presenteeism multiplied by 22 working days per month then multiplied by 12 months per year. The computed economic costs was converted to USD based on the conversion rate from the Central Bank of the Philippines during the study period [16].

### Data analysis

Descriptive statistics were reported for overall MIDAS Score and MIDAS Grade, SF-36 quality of life score across each of the eight domains, workplace triggers, coping mechanisms, health care utilization, assistance in the workplace, and economic costs. MIDAS Grade was dichotomized between high migraine disability (MIDAS III/IV) and low migraine disability (MIDAS I/II). Chi-square tests and student *t*-tests were done to determine differences in demographic information and migraine disability.

To determine predictors of migraine disability, adjusted odds ratios (OR) and their 95% confidence intervals (CI) were calculated for high vs. low migraine disability for the following socio-demographic factors: gender, age, educational attainment, employee rank, and gross monthly income from the company. The final multiple logistic regression model included all covariates except gross monthly income given its collinearity with employee rank.

Migraine disability (high/low) was used as a predictor for SF-36 quality of life score, adjusted for gender, age, educational attainment, and employee rank using multiple linear regression, and with health care utilization adjusted for the same covariates using multiple logistic regression. Finally, given that not all migraine positive screens reported absenteeism and presenteeism, a two-part model was used to determine the economic costs of migraine between low and high migraine disability: the first part was a logistic regression model that determined the probability of reporting any economic cost of migraine, while the second part was a generalized linear model (GLM) fit with a log link function and gamma (for absenteeism) or Poisson distribution (for presenteeism and total days and cost lost to migraine), with the function and distribution confirmed using the box-cox test and modified Park test, respectively [17]. All analyses were conducted using Stata IC 15.1 (StataCorp, College Station, TX).

## Results

A total of 954 respondents provided consent and attempted to respond to the survey, or a response rate of around 4.1%. Of those, 511 screened positive for migraine, with 193 reported they have been diagnosed with migraine by a licensed medical professional and the rest screening positive using the ID Migraine™ Test. Of those who screened positive with the ID Migraine™ Test, 270 (84.9%) reported symptoms of nausea, 282 (88.7%) reported the headaches limited their ability to do daily tasks, and 284 (89.3%) reported that light was bothering them when they had headaches. MIDAS Grade was available for 372 positive migraine screens, with 100 (26.9%) reporting Grade I, 64 (17.2%) reporting Grade II, 93 (25.0%) reporting Grade III, and 115 (30.9%) reporting Grade IV (Fig. 1).

Table 1 summarizes the demographic information of positive migraine screens, disaggregated by migraine disability severity. Two in three (67.5%) were female, around half (48.9%) were from the ages of 25 to 34 years old, four in five (80%) were college graduates, around half (53.2%) were rank and file employees, and about two in five (36.6%) earned less than PHP20 000 (USD400) monthly.

**Table 1:** Demographics of positive migraine screens and association with high migraine disability (*n* = 511)

	Overall (*n* = 511)	Low migraine disability (MIDAS I/II) (*n* = 164)	High migraine disability (MIDAS III/IV) (*n* = 208)	***p***-value	Adjusted odds ratio (95% CI)
**Females**, *n* (%)	345 (67.5%)	94 (57.3%)	142 (68.2%)	0.029	1.60 (1.03–2.49)
**Age**, mean (SD)	31.6 (7.5)	32.3 (7.7)	31.5 (7.3)	0.287	0.98 (0.96–1.02)
**Finished college**, *n* (%)	406 (79.4%)	140 (85.3%)	156 (75.0%)	0.014	0.55 (0.31–0.96)
**Employee rank,** *n* (%)				0.293	
Rank and file	272 (53.2%)	79 (48.2%)	117 (56.2%)		ref.
First-level management	139 (27.2%)	50 (30.5%)	52 (25.0%)		0.91 (0.54–1.53)
Middle & senior management	100 (18.6%)	35 (21.3%)	39 (18.8%)		0.84 (0.47–1.50)
**Gross monthly income (PHP)**, *n* (%)				0.193	*(not considered in final regression model due to collinearity with employee rank)*
Less than 20,000	187 (36.6%)	53 (32.3%)	74 (35.6%)		
20,000 to 29,999	166 (32.5%)	50 (30.5%)	76 (36.5%)		
30,000 to 39,999	62 (12.1%)	22 (13.4%)	26 (12.5%)		
40,000 and above	158 (18.8%)	39 (23.8%)	32 (15.4%)		

Notes: 1. Models were adjusted for gender, age, educational attainment, and employee rank; 2. PHP: Philippine Pesos, Conversion rate approximately PHP50 = USD1; 3. MIDAS Grade data available for 372 of 511 positive migraine screens

Between low and high migraine disability, differences in demographics were apparent in females, where a higher proportion of females was reported among those with high migraine disability (68.2%) than low migraine disability (57.3%, *p* = 0.029). The reverse was true for college graduates (75.0% for high v 85.3% for low, *p* = 0.014). These relationships remained after adjusting for age and employee rank. Females had 1.6 times the odds of having high disability migraine (95% CI: 1.03–2.49) than males, while college graduates had around half the odds of having high disability migraine (0.55, 95% CI: 0.31–0.96) than non-college graduates. Age, employee rank, and gross monthly income were not associated with high migraine disability (Table 1).

**Fig. 2:**
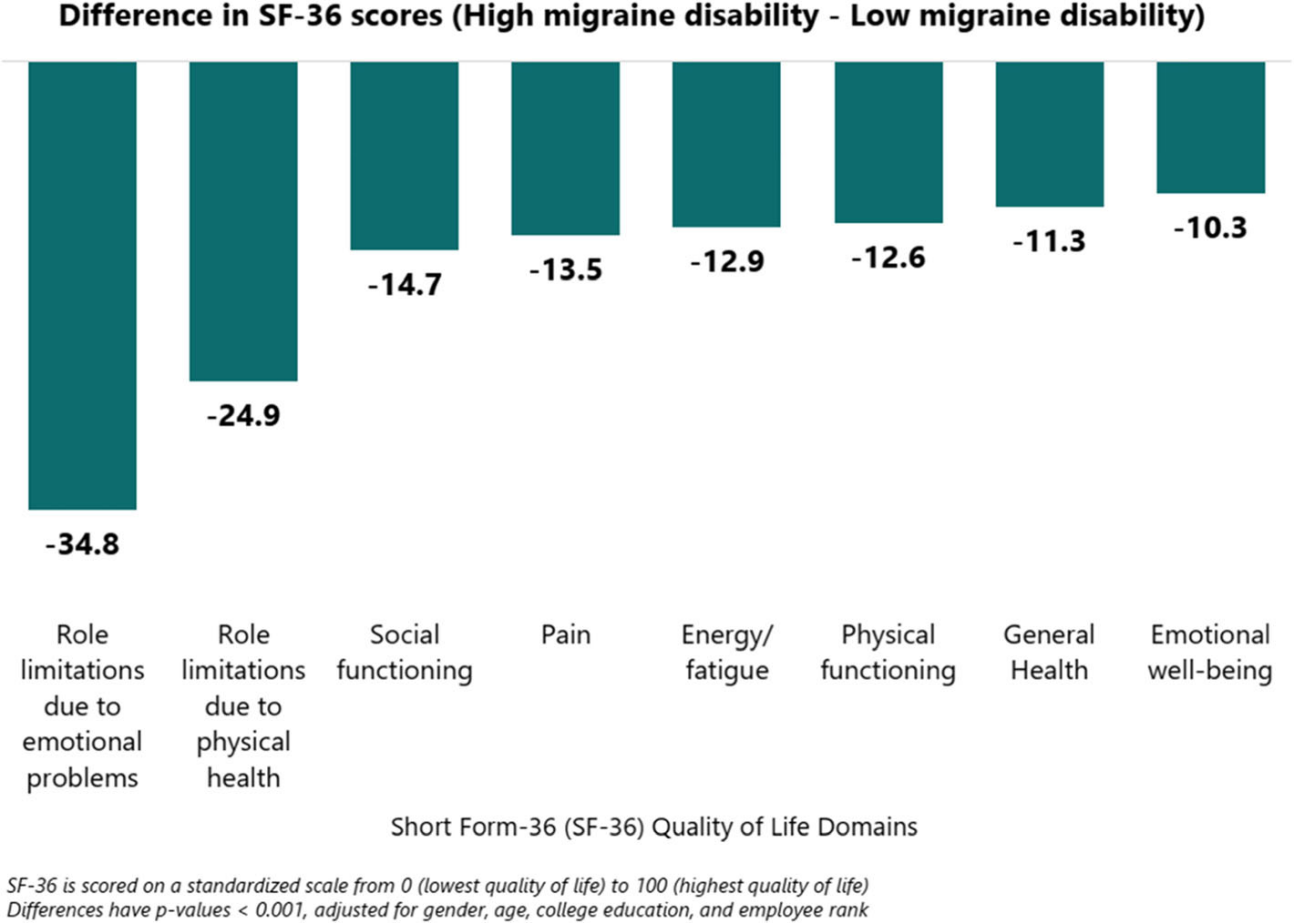
Adjusted differences in Short Form-36 (SF-36) quality of life scores between low and high migraine disability. Notes: Models were adjusted for gender, age, educational attainment, and employee rank

Quality of life was significantly lower among those with high migraine disability than those with low migraine disability across all eight SF-36 domains, after adjusting for gender, age, educational attainment, and employment rank (Fig. 2). Quality of life scores were at least 10 points lower among those with high migraine disability, out a total score of 100. The two highest differences were found in role limitations, both physically and mentally (− 34.8 and − 24.9, respectively, *p* < 0.0001).

**Fig. 3:**
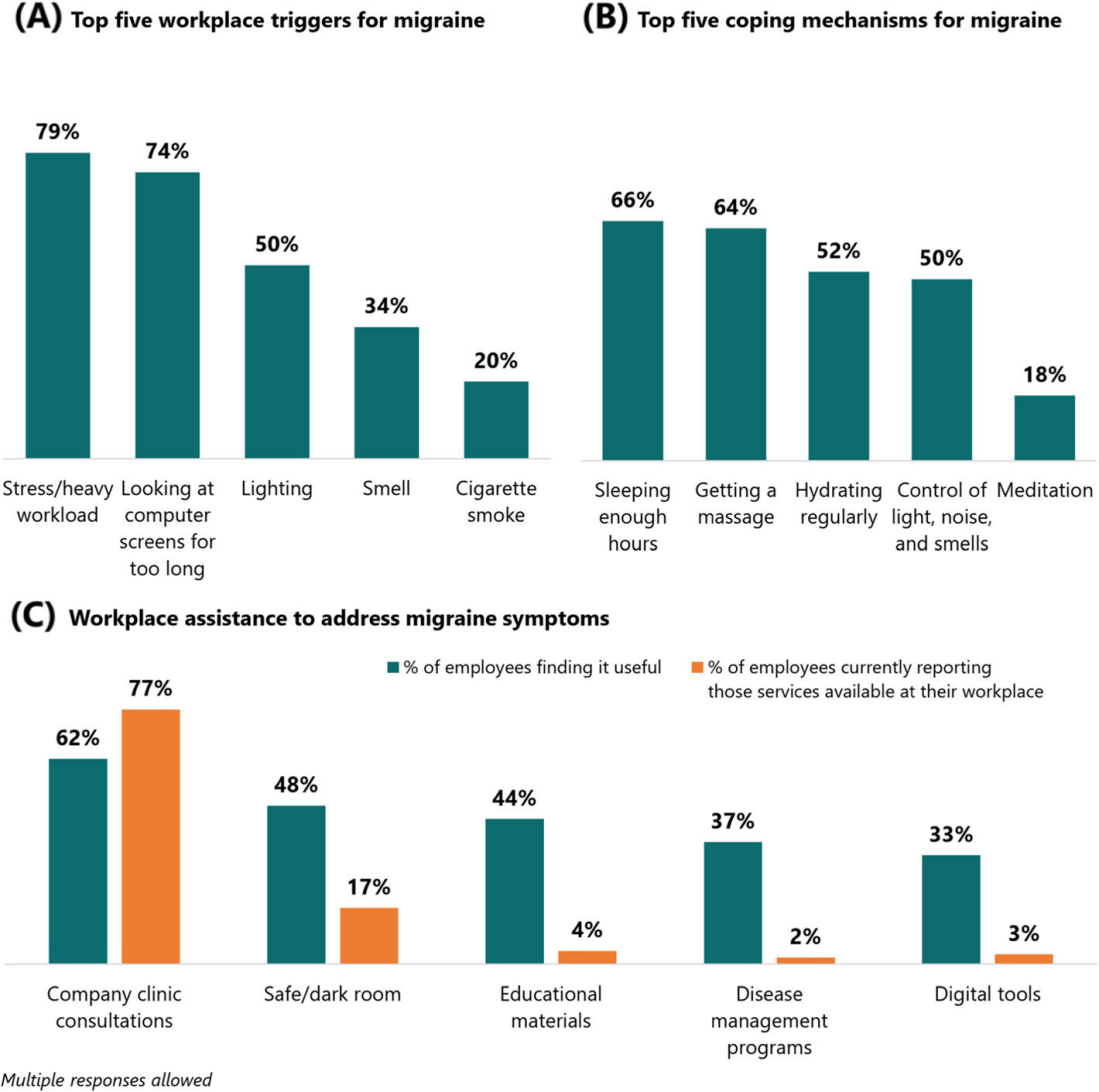
Workplace conditions and support for migraine

Around one in five respondents (21.6%) reported worrying often or constantly about the next headache happening in the workplace. About 72 % (72.7%) reported worrying sometimes about the next headache while only 5.7% did not report worrying about the next headache. Respondents identified multiple workplace triggers, the top two of which were stress/heavy workload and looking at computer screens for too long (Fig. 3, panel A). They also identified multiple coping mechanisms, such as sleeping enough hours and getting a massage (Fig. 3, panel B). Only 1.7% did not identify any triggers, while only 4.9% did not identify any coping mechanism.

Around two in three (63.8%) were open about discussing their migraine condition to others. More than half (55.8%) reported that their workmates were aware of their condition, while two in five (43.1%) reported that their supervisor was aware of their condition.

In terms of support provided by employers for migraine, three-fourths (76.9%) reported having company clinics available at their workplace, with about two-thirds of all respondents (61.5%) finding those consultations useful (Fig. 3, panel C). Only 17.2% of all respondents reported having a safe/dark room, but about half (48.3%) of all respondents found having a safe/dark room useful in the workplace. A safe/dark room is a separate quiet room available in some workplaces to serve as a place for employees to rest especially at the onset of a migraine attack. Similar discrepancies in proportions were found for other support programs, such as educational materials about migraine, disease management programs, and digital tools such as symptom tracking apps.

**Fig. 4:**
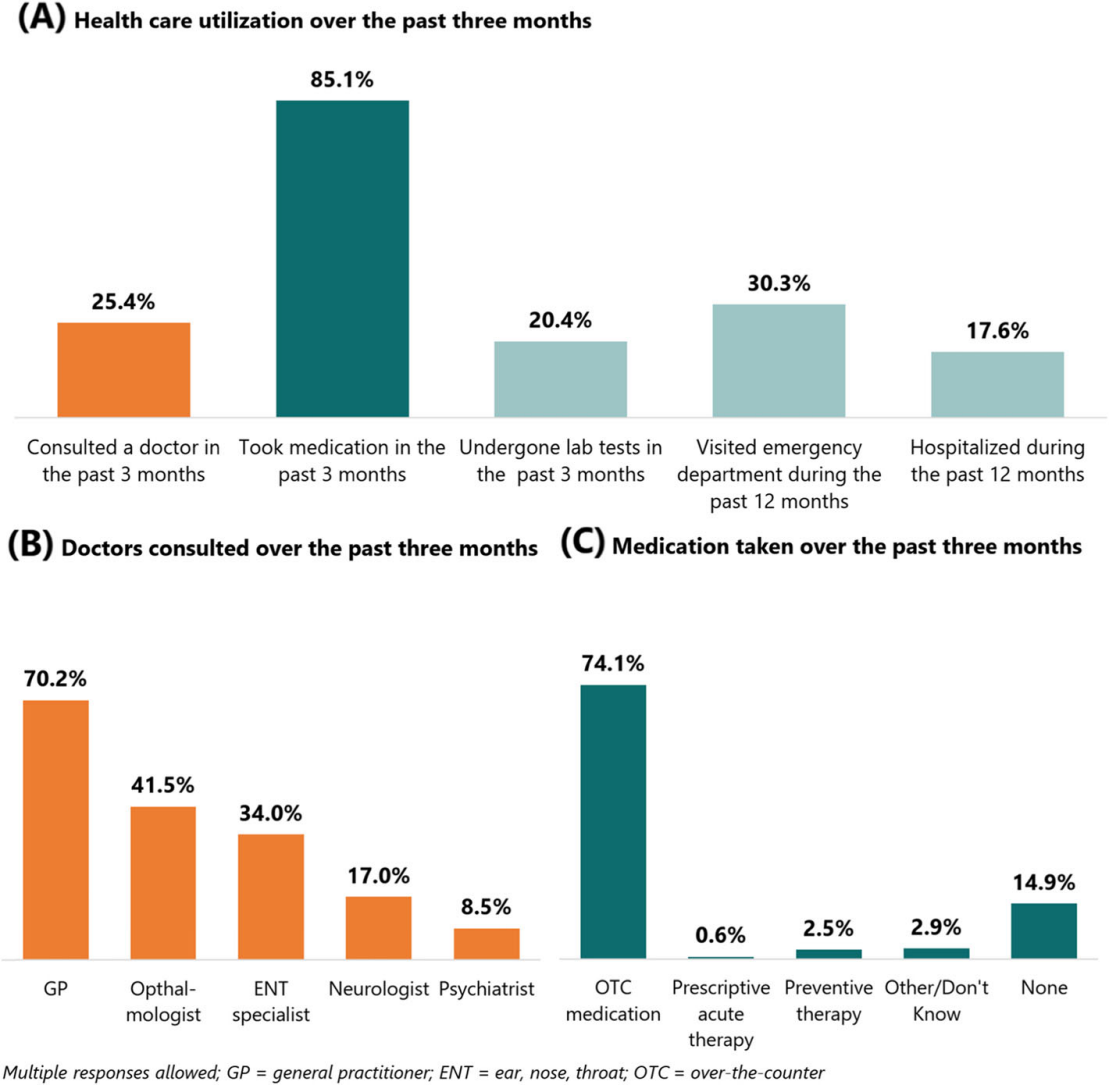
Health care utilization for migraine-related services

In the past three months, about two in three (61.8%) reported using their company-provided health insurance and medicines allowance benefits to pay for migraine-related services. Of those, one in four (25.4%) reported having outpatient consults with a physician regarding migraine-related symptoms (Fig. 4, panel A). Of those, 70.2% sought a general practitioner (GP) at least once, 41.5% sought an ophthalmologist at least once, 34.0% an ear, nose, throat (ENT) specialist at least once, and 17.0% a neurologist at least once (Fig. 4, panel B). Five in six (85.2%) reported taking some form of medication, majority of which were over-the-counter medications, e.g. paracetamol, ibuprofen (74.1%). Only a few respondents reported taking prescriptive acute therapy, e.g. triptans (0.6%) and preventive therapy, e.g. atenolol (2.5%) (Fig. 4, panel C). Finally, one in five (20.4%) reported having undergone laboratory tests in the past three months, one in three (30.3%) reported visiting the hospital emergency room for headache symptoms in the past 12 months, and one in six (17.6%) reported being hospitalized for a migraine emergency in the past 12 months.

Migraine disability was significantly associated with some health care utilization variables. Those with high migraine disability were more likely than those with low migraine disability to consult a doctor within the past three months (OR: 3.11, 95% CI: 1.78–5.43), undergo a laboratory test in the past three months (OR: 1.84, 95% CI: 1.03–3.27), and visit the emergency department during the past 12 months (OR: 2.09, 95% CI: 1.25–3.48) (Table 2).

**Table 2:** Associations of health care utilization and workplace productivity losses with migraine disability (high or MIDAS III/IV vs low or MIDAS I/II)

	**Odds ratio**	**Standard error**	***p*-value**	**95% confidence interval**
**Health care utilization**				
Consulted a doctor in the past 3 months	3.11	0.88	< 0.0001	1.78–5.43
Took medication in the past 3 months	1.67	0.58	0.137	0.85–3.30
Undergone lab tests in the past 3 months	1.84	0.54	0.038	1.03–3.27
Visited emergency department during the past 12 months	2.09	0.54	0.005	1.25–3.48
Hospitalized during the past 12 months	1.85	0.60	0.058	0.98–3.47
**Workplace productivity losses**				
*Logistic regression (part 1) of two-part model*				
Reported at least 1 day of absenteeism in the past 3 months	6.63	1.65	< 0.0001	4.07–10.81
Reported at least 1 day of presenteeism in the past three months	2.49	0.34	< 0.0001	1.83–3.15
Reported at least 1 day of absenteeism or presenteeism in the past 3 months	2.73	0.41	< 0.0001	1.93–3.54
*Generalized linear model with log link family and gamma distribution for absenteeism and Poisson distribution for presenteeism and total workdays lost to migraine (part 2) of two-part model*				
	**Marginal effect**	**Standard error**	***p*-value**	**95% confidence interval**
Number of days of absenteeism in the past 3 months	2.67	0.33	< 0.0001	2.04–3.31
Number of days of presenteeism in the past 3 months	5.08	0.31	< 0.0001	4.48–5.69
Total number of days lost due to migraine in the past 3 months	8.08	0.39	< 0.0001	7.31–8.86
Monthly costs due to absenteeism (PHP)	974.3	118.3	< 0.0001	742.5–1206.1
Monthly costs due to presenteeism (PHP)	1889.4	39.0	< 0.0001	1813.0–1965.8
Total monthly costs due to migraine (PHP)	2982.2	52.3	< 0.0001	2879.7–3084.6

Notes: 1. Models were adjusted for gender, age, educational attainment, and employee rank; 2. Workplace productivity losses were assessed using a two-part model, where the first part measured the likelihood of reporting at least a day lost to migraine, and the second part measuring the number of days and associated costs lost due to migraine for those reporting at least a day lost to migraine; 3. PHP: Philippine Pesos, Conversion rate approximately PHP50 = USD1

On average, those with low migraine disability reported around two days affected by migraine over a three month period (1.92, 95% CI: 1.61–2.24) and nine days or more than four times more for those with high migraine disability (8.97, 95% CI: 8.49–9.46) (Table 3). Monthly costs on average lost due to migraine amounted to PHP2 316.2 (USD46) (95% CI: 2232.9–2399.5). Costs significantly differed between migraine disability: monthly costs lost due to migraine for those with low disability averaged around PHP865.1 (USD17) (95% CI: 754.3–975.9) while costs quadrupled for those with high disability at PHP3 440.8 (USD69) (95% CI: 3323.4–3558.3). These costs, when annualized, translate to approximately PHP27 794.4 (USD556) per person for everyone with migraine, PHP10 381.2 (USD207) per person for those with low migraine disability, and PHP41 289.6 (USD826) per person for those with high migraine disability.

**Table 3:** Marginal effects of work productivity losses and economic costs due to migraine

	Low migraine disability (95% CI)	High migraine disability (95% CI)
Number of days of absenteeism in the past 3 months	0.70 (0.50–0.89)	3.20 (2.75–3.65)
Number of days of presenteeism in the past 3 months	1.24 (0.99–1.48)	5.75 (5.36–6.14)
Total number of days lost due to migraine in the past 3 months	1.92 (1.61–2.24)	8.97 (8.49–9.46)
Monthly costs due to absenteeism (PHP)	263.6 (187.9–339.4)	1151.1 (984.7–1317.6)
Monthly costs due to presenteeism (PHP)	605.6 (514.9–696.3)	2279.6 (2185.3–2373.9)
Total monthly costs due to migraine (PHP)	865.1 (754.3–975.9)	3440.8 (3323.4–3558.3)

Notes: Adjusted for gender, age, educational attainment, and employee rank; workplace productivity losses were assessed using a two-part model, where the first part measured the likelihood of reporting at least a day lost to migraine, and the second part measuring the number of days and associated costs lost due to migraine for those reporting at least a day lost to migraine; PHP: Philippine Pesos, Conversion rate approximately PHP50 = USD1

## Discussion

This study provided a comprehensive view of how migraine affects employees in selected workplaces in the Philippines. As much as nine days of work may be affected over a three-month period, or about 10% of all employee work hours, for those with the most severe forms of migraine. This finding is in the middle of previously reported estimates in other contexts. This is higher than the mean of nine days for Malaysia [12], but lower than the 45 days reported in the United States over the same time period [18]. A systematic review in 2011 found the range of workdays missed ranges from 0.23 to 16.4 across multiple studies [7].

On the other hand, the economic costs lost due to migraine was much lower than previously reported in upper middle income and upper income countries, where estimates ranged upwards of USD1 000 per employee annually [6, 10, 12]. In this study, the mean annual cost was at USD500 per employee for those with low migraine disability and USD800 per employee for those with high migraine disability. This is because majority of the respondents in the study only had monthly incomes of less than USD600, which represents the median household income in the country [19], but much lower than median household incomes of richer countries. Regardless, relative to household income, these costs remain significant to Filipino employees and their families.

Presenteeism played a bigger role than absenteeism in workplace productivity losses, reflecting results from other countries [12, 20]. In this study, those with high migraine disability were only expected to take a sick leave for about 12 days a year due to migraine. Philippine labor law does not guarantee paid sick leave, but instead requires a minimum of five days of paid leave for whatever reason, although in practice employees receive more days of paid leave as a function of company benefits or collective bargaining agreements [21]. Therefore, this does not fully explain why absenteeism was less prevalent than presenteeism especially that the companies participating in the study provided generous company benefits such as more days of paid sick leave. Other studies did not provide potential explanations for this as well [12, 20], and further research is needed to understand the motivating factors behind why those with migraine choose to work despite experiencing symptoms that affect their work productivity. Regardless, higher rates of presenteeism may reflect worsening of migraine over the long term, and exacerbate to stress, which was cited as the top migraine workplace trigger, and introduce a positive feedback loop of stress and migraine that will only increase economic losses and poorer quality of life over time [22].

There are many opportunities to address migraine better in the workplace, and the companies selected in this sample have already implemented some. Since all employees in the sample had generous company-provided private health insurance and medicine allowances, health care utilization rate for migraine was higher than the national health care utilization rate for any type of service, which was 8% based on the 2017 Demographic and Health Survey [23]. The types of doctors sought for consultation also reflected this high company benefit utilization rate, since GPs are readily accessible in the in-house clinics of these companies, which are required by occupational health and safety laws in the Philippines, and neurologists were not commonly sought since services by neurologists are not covered by private health insurance plans. The frequency of ophthalmology consultations and desire for a safe/dark room in the workplace is reflective of prolonged computer usage, which was cited as one of the top triggers for migraine.

The high intake of medicines may be a function of utilization of company medicine allowances, and some companies also subscribe to pharmacy benefit managers that handle bulk medicine fulfillment based on a set formulary. However, use of prescriptive acute and preventive therapies remain very low, and this may be because employees do not receive enough information on migraine. About two in five employees with migraine would like more educational materials and migraine disease management programs available in their workplace, but currently less than 5% of employees report these being available. Health education on migraine may present opportunities to introduce holistic prevention and wellness, as well as alternative treatment options, such as preventive medications, for migraine that provide more than symptom relief for headaches.

Additionally, disease management programs should be tailored towards specific socio-demographic groups. This study did not find significant associations between age and migraine disability, which was more apparent in other studies [1, 2]. This is likely because the study sample comprised of younger adults, and this is expected given the current median age of the Philippines is around 24 years old [24]. On the other hand, females were more likely to have high migraine disability than males, which was consistent with results from other studies [1, 2, 7, 12]. Other studies have hypothesized that there may be biological factors, sociological factors and health-seeking behavioral factors involved [6, 11], but further research is needed to understand these differences in the Philippine context. Finally, higher education seemed to be a protective risk against high migraine disability, a finding also seen in other studies. These studies hypothesized that education may be a proxy indicator for lifestyle and socioeconomic status, which is associated with increased exposure to migraine triggers outside the workplace such as heavy traffic during commute and poor living conditions [25, 26]. This hypothesis may also be supported by the study’s results, since those with high migraine disability had the greatest difference in quality of life score on domains related to physical and emotional role limitations.

Altogether, an economic case for more expensive forms of migraine prevention and treatment may be built based on the results of this study. Given that the workplace burden is significant and there are companies willing to provide generous health benefits for their employees, treatments may be justified on the expected savings from productivity losses. However, further economic research is needed to gauge the willingness to pay of these companies to integrate those treatments in their medicine allowance formulary or private health insurance plans, and any migraine treatment must also be assessed on factors such as efficacy and cost-effectiveness. Employers must also be educated on the burden and impact of migraine in the workplace, and results from this study may begin that important conversation for the benefit of employees and companies overall.

### Limitations

The study had some limitations. First, the sample is not representative of all workplaces in the Philippines given the selection criteria and willingness of employers to participate in the study. The researchers tried to address this by reaching out to more than 100 companies to participate in the survey, with 12 eventually included in the final sample. Non-response bias among the selected companies could also not be assessed, as the researchers did not have access to company employee demographic information that could be used for weighting and adjustment for bias. Second, some companies did not want to be identified even with their industry, so analyses could not be stratified by industry. Third, the variables were all self-reported by the respondent, so a definitive migraine diagnosis was not made in this study, and other variables such as health care utilization history could not be independently verified because all responses were anonymized. Nonetheless, this study provided meaningful data for the selected industries and may inform initiatives and programming for migraine health care.

## Conclusion

Migraine poses a significant burden to employee productivity in the Philippines, with annualized costs due to migraine costing as much as PHP40 000 (USD826) per person for those with high migraine disability and PHP10 000 (USD200) per person for those with low migraine disability. Quality of life was significantly lower among those with high migraine disability than those with low migraine disability across all eight SF-36 domains. The two highest differences were found in role limitations, both physically and mentally. There are companies in the Philippines that seems to provide ample support for medical consultations through their employee benefits programs, but there are opportunities to implement other interventions such as prevention and wellness programs. Most medication taken for migraine remain OTC medicines for symptom relief, and further research is needed to gauge willingness of employers to pay for preventive medication for migraine.

## Data Availability

The datasets generated and/or analyzed during the current study are not publicly available due to anonymity agreements between the authors and the participating companies, but some may be made available from the corresponding author on reasonable request.

## Abbreviations

ENT: ear, nose, throat
CI: confidence interval
GLM: generalized linear model
GP: general practitioner
MIDAS: Migraine Disability Assessment
OR: odds ratio
OTC: over-the-counter
PHP: Philippine Pesos
SF-36: Short Form-36 Questionnaire
SMS: short messaging service
USD: United States Dollars

## References

[1] Woldeamanuel YW, Cowan RP (2017). Migraine affects 1 in 10 people worldwide featuring recent rise: a systematic review and meta-analysis of community-based studies involving 6 million participants. J Neurol Sci.

[2] Stovner LJ, Nichols E, Steiner TJ, Abd-Allah F, Abdelalim A, Al-Raddadi RM (2018). Global, regional, and national burden of migraine and tension-type headache, 1990–2016: a systematic analysis for the global burden of disease study 2016. Lancet Neurol.

[3] Stark RJ, Ravishankar K, Siow HC, Lee KS, Pepperle R, Wang S-J (2013). Chronic migraine and chronic daily headache in the Asia-Pacific region: a systematic review. Cephalalgia..

[4] Roxas A, Gose M, Dominguez J, Liban S, Rosales R, Sosa MG (2007). The Prevalences of stroke, parkinsonism, dementia, migraine and epilepsy in the Philippines - part 2: application of the PNA questionnaire in the 2003 National Nutrition Health Survey. Philipp J Neurol.

[5] Institute of Health Metrics and Evaluation. Philippines Global Burden of Disease 2017 [Internet]. Seattle WA: IHME; 2020 [cited 2020 Oct 9]. Available from: http://www.healthdata.org/philippines

[6] Agosti R (2018). Migraine burden of disease: from the Patient’s experience to a socio-economic view. Headache J Head Face Pain..

[7] Lantéri-Minet M, Duru G, Mudge M, Cottrell S (2011). Quality of life impairment, disability and economic burden associated with chronic daily headache, focusing on chronic migraine with or without medication overuse: a systematic review. Cephalalgia Int J Headache.

[8] Freitag FG (2007). The cycle of migraine: patients’ quality of life during and between migraine attacks. Clin Ther.

[9] Leso V, Gervetti P, Mauro S, Macrini MC, Ercolano ML, Iavicoli I. Shift work and migraine: A systematic review. J Occup Health. 2020 [cited 2020 Jul 23];62(1). Available from: https://www.ncbi.nlm.nih.gov/pmc/articles/PMC7154593/10.1002/1348-9585.12116 PMC7154593 32515906

[10] Linde M, Gustavsson A, Stovner LJ, Steiner TJ, Barré J, Katsarava Z (2012). The cost of headache disorders in Europe: the Eurolight project. Eur J Neurol.

[11] Stang P, Cady R, Batenhorst A, Hoffman L (2001). Workplace productivity. A review of the impact of migraine and its treatment. PharmacoEconomics..

[12] Wong LP, Alias H, Bhoo-Pathy N, Chung I, Chong YC, Kalra S (2020). Impact of migraine on workplace productivity and monetary loss: a study of employees in banking sector in Malaysia. J Headache Pain.

[13] Cousins G, Hijazze S, de Laar FAV, Fahey T (2011). Diagnostic accuracy of the ID migraine: a systematic review and meta-analysis. Headache J Head Face Pain..

[14] Csépány É, Tóth M, Gyüre T, Magyar M, Bozsik G, Bereczki D (2018). The validation of the Hungarian version of the ID-migraine questionnaire. J Headache Pain..

[15] de Mattos ACMT, de Souza JA, Moreira Filho PF, Jurno ME, Velarde LGC, de Mattos ACMT (2017). ID-migraine™ questionnaire and accurate diagnosis of migraine. Arq Neuropsiquiatr.

[16] Bangko Sentral ng Pilipinas. Online Statistical Interactive Database. 2016 [cited 2016 Oct 20]. Available from: http://www.bsp.gov.ph/statistics/statistics_online.asp

[17] Deb P, Norton EC (2018). Modeling health care expenditures and use. Annu Rev Public Health.

[18] Munakata J, Hazard E, Serrano D, Klingman D, Rupnow MFT, Tierce J (2009). Economic burden of transformed migraine: results from the American Migraine Prevalence and Prevention (AMPP) study. Headache J Head Face Pain.

[19] Philippine Statistics Authority. Annual Family Income is Estimated at PHp 313 Thousand, on Average, in 2018 [Internet]. Manila, Philippines: Philippine Statistics Authority; 2019 Dec. Report No.: 2019–206. Available from: https://psa.gov.ph/income-expenditure/fies/title/Annual%20Family%20Income%20is%20Estimated%20at%20%20PhP%20313%20Thousand%2C%20on%20Average%2C%20In%202018

[20] Brooks A, Hagen SE, Sathyanarayanan S, Schultz AB, Edington DW (2010). Presenteeism: critical issues. J Occup Environ Med.

[21] de Vera AF. The labor code of the Philippines, 2017 Edition. Manila: LexisNexis; 2018

[22] Aronsson G, Gustafsson K, Dallner M (2000). Sick but yet at work. An empirical study of sickness presenteeism. J Epidemiol Community Health.

[23] ICF International, Philippine Statistics Authority (2018). National Demographic and Health Survey 2017.

[24] Philippine Statistics Authority. Philippine Population Surpassed the 100 Million Mark (Results from the 2015 Census of Population). Manila, Philippines; 2017 Jun. Report No.: 2017–148. Available from: https://psa.gov.ph/population-and-housing/node/120080

[25] Rees DI, Sabia JJ (2011). The effect of migraine headache on educational attainment. J Hum Resour.

[26] Le H, Tfelt-Hansen P, Skytthe A, Kyvik KO, Olesen J (2011). Association between migraine, lifestyle and socioeconomic factors: a population-based cross-sectional study. J Headache Pain..

